# Sinomenine attenuates pulmonary fibrosis by downregulating TGF-β1/Smad3, PI3K/Akt and NF-κB signaling pathways

**DOI:** 10.1186/s12890-024-03050-5

**Published:** 2024-05-10

**Authors:** Fuqiang Yao, Minghao Xu, Lingjun Dong, Xiao Shen, Yujie Shen, Yisheng Jiang, Ting Zhu, Chu Zhang, Guangmao Yu

**Affiliations:** 1 Department of Thoracic Surgery, Shaoxing People’s Hospital, Shaoxing, Zhejiang China; 2 School of Medicine, ShaoXing University, Shaoxing, Zhejiang China

**Keywords:** Pulmonary fibrosis, Extracellular matrix, Sinomenine, TGF-β1/Smad3, PI3K/Akt, NF-κB

## Abstract

**Background:**

Since COVID-19 became a global epidemic disease in 2019, pulmonary fibrosis (PF) has become more prevalent among persons with severe infections, with IPF being the most prevalent form. In traditional Chinese medicine, various disorders are treated using Sinomenine (SIN). The SIN’s strategy for PF defense is unclear.

**Methods:**

Bleomycin (BLM) was used to induce PF, after which inflammatory factors, lung histological alterations, and the TGF-/Smad signaling pathway were assessed. By administering various dosages of SIN and the TGF- receptor inhibitor SB-431,542 to human embryonic lung fibroblasts (HFL-1) and A549 cells, we were able to examine proliferation and migration as well as the signaling molecules implicated in Epithelial-Mesenchymal Transition (EMT) and Extra-Cellular Matrix (ECM).

**Results:**

In vivo, SIN reduced the pathological changes in the lung tissue induced by BLM, reduced the abnormal expression of inflammatory cytokines, and improved the weight and survival rate of mice. In vitro, SIN inhibited the migration and proliferation by inhibiting TGF-β1/Smad3, PI3K/Akt, and NF-κB pathways, prevented the myofibroblasts (FMT) of HFL-1, reversed the EMT of A549 cells, restored the balance of matrix metalloenzymes, and reduced the expression of ECM proteins.

**Conclusion:**

SIN attenuated PF by down-regulating TGF-β/Smad3, PI3K/Akt, and NF-κB signaling pathways, being a potential effective drug in the treatment of PF.

**Supplementary Information:**

The online version contains supplementary material available at 10.1186/s12890-024-03050-5.

## Introduction

The appearance of the severe acute respiratory syndrome coronavirus type 2 (SARS-CoV-2) in Wuhan in December 2019 caused more than 641 million cases of severe acute progressive respiratory diseases worldwide and more than 6.6 million deaths by December 2022 [1]. Even though it has been four years since the outbreak of New Crown Pneumonia, people nowadays often live with a high incidence of influenza, during which time hospital emergency centers are filled with many patients who test positive for New Crown infections. Some infected people are still prone to develop interstitial pneumonia, and even life-threatening acute respiratory distress syndrome (ARDS) [2]. ARDS is characterized by excessive inflammation and endothelial dysfunction, and a number of specific inflammatory factors and markers of endothelial dysfunction have been shown to correlate with disease extent and mortality in COVID-19 [3, 4]。For the published histopathological analysis of COVID-19 postmortem lungs the literature shows Idiopathic pulmonary fibrosis (IPF) manifestations in addition to diffuse alveolar damage and hyaline membranes [5]。IPF is the most common form of PF, with a median survival time of 2–4 years [6]. The only feasible treatment for patients with end-stage PF is still lung transplantation due to the lack of appropriate drug treatments. At present, the drugs for treating PF have side effects on the liver, skin, myocardium, and growth and development of organism which limit their use, especially in patients with liver problems [7]. Therefore, it is of the utmost importance to find new drugs with improved therapeutic effects and fewer side effects.

PF is considered an inflammatory repair mechanism of repeated micro-injury of the alveolar epithelium, which leads to the directional chemotaxis of fibrogenic mediators, specifically activating fibroblasts to differentiate into myofibroblasts and producing extracellular matrix (ECM). The excessive accumulation of ECM destroys the structure of the normal lung tissue, affects gas exchange, and the ECM-cell signaling induces positive feedback in ECM production, which further aggravates the progress of fibrosis [8, 9].

Although the mechanism of PF is not clear at present, many hypotheses have been formulated to explain its mechanism, and among these, that of EMT of alveolar epithelial cells is one of the most substantial. Nuclear factor κB (NF-κB) is an important mediator of EMT. It promotes the transcription of various inflammatory cytokines, such as tumor necrosis factor α (TNF-α), interleukin (IL), and transforming growth factor β (TGF-β), which are strongly related to the progress of IPF, especially TGF-β [10–12]. The classical pathway of TGF-β-mediated fibrosis is related to the Smad protein family signaling pathway. Once the downstream effector complex enters the nucleus, it regulates the expression of EMT-related target genes. However, also non-Smad pathways exist downstream of TGF-β [13]. PI3K/Akt pathway is the key signal node in the process of fibrosis, and it interacts with the TGF-β/Smad signal transduction pathway to alleviate the progress of PF [14, 15]. Therefore, these factors should be considered when evaluating the effect of drugs on IPF.

Sinomenine (SIN) is an alkaloid monomer extracted from the dried stems of *Sinomenium actum* Rehd.et wils., and is a popular traditional Chinese medicine used to treat rheumatoid arthritis and arrhythmia. Recent studies show that SIN is effective against tumor and inflammatory diseases. It inhibits the migration and invasion of breast cancer cells, prostate cancer cells, and glioblastoma cells [16–18], as well as oxidative stress, inflammation, and apoptosis to alleviate acute liver injury [19]. Moreover, SIN alleviates liver fibrosis and improves airway remodeling caused by chronic asthma by regulating TGF-β/Smad in vitro and in vivo [20, 21]. However, the anti-fibrosis mechanism of action of SIN on PF is unclear.

This study showed that SIN alleviated the pathological changes in the lung induced by bleomycin (BLM), reduced the abnormal expression of inflammatory cytokines, and improved the weight and survival rate of mice treated with BLM. In vitro experiments showed that SIN inhibited the migration and proliferation of human embryonic lung fibroblast cell line (HFL-1) and human lung adenocarcinoma basal epithelial cell line (A549) cells by the inhibiting TGF-β1/Smad3, PI3K/Akt, and NF-κB pathways, prevented the transformation of HFL-1 into myofibroblasts (FMT), reversed the EMT of A549 cells, restored the balance of matrix metalloenzymes, and reduced the expression of ECM marker proteins. These results support the role of SIN as a potential medicine for IPF treatment.

## Materials and methods

### BLM-induced IPF mouse model

A total of 36 male C57BL-6 J mice (8–10 weeks, 25–28 g) (SCXK(Zhe)2022-0005) were purchased from Hangzhou Qizhen Laboratory Animal Technology Co., Ltd. (Hangzhou, China), and were housed under controlled environmental conditions (22 ℃ and 12:12-hour light-dark cycle). A total of 36 mice were randomly allocated into 6 groups (6 mice per group): Control group, BLM group, B + L group (SIN 50 mg/kg), B + M group (SIN 100 mg/kg), B + H group (SIN 150 mg/kg) and SIN group (SIN 150 mg/kg). Except Control and SIN group, PF was induced with 3 mg/kg bleomycin (BLM) (HY-17,565; MCE, Monmouth Junction, NJ, USA) preparation solution via Intratracheal injection on the first day. The mice were injected with SIN or 0.9% NaCl solution (control group) intraperitoneally for two weeks after day 1 of modeling. The body weight was monitored and recorded every day, and the mice were euthanized by injecting excessive pentobarbital sodium (150 mg/kg) on the 14th day.

### Cell culture and reagents

HFL-1 and A549 were purchased from the cell bank of China Academy of Sciences (Shanghai, China). All the cells used were passaged less than 20 times. Both cell types were cultured in Ham’s F12K medium (Bio-Channel, Nanjing, China) supplemented with 10% fetal bovine serum (FBS) (Gibco, GrandIsland, NY, USA), and incubated in a humid environment of 37 ℃ and 5% CO2. When the cells reached 70–80% confluence, they were put into experiments. All the experiments were carried out in triplicate. Moreover, HFL-1 and A549 cells in each group were starved in serum-free F12K for 12 h, then 10 ng/mL TGF-β1 (PeproTech, Cranbury, NJ, USA) was added for 12 h to induce fibrosis, and then the cells were treated with SIN (S235903, Selleck, Shanghai, China) for 12 h. A stock solution of SIN was prepared (solvent: water) at a concentration of 1 mM, stored at -20 ℃, and diluted to the specified working solution before each experiment. The TGF-β receptor inhibitor SB-431,542 (MCE, Monmouth Junction, NJ, USA) was also used to further explore the mechanism of fibrosis in the cell model, and starved cells were treated with the inhibitor 30 min before TGF-β1 treatment.

### Determination of drug toxicity and cell proliferation rate

HFL-1 and A549 cells were seeded into 96-well plates (100 µL/well, NEST, Wuxi, China) at a density of 8,000–10,000 cells per well and cultured overnight. When the cells reached 70–80% confluence, they were treated with different concentrations of SIN for 12 h and 24 h. Cell viability was assessed by Cell counting kit − 8 (CCK-8; MCE, Monmouth Junction, NJ, USA) after the addition of 10 µL/well reagent and incubated for 1.5 h. In addition, A549 and HFL-1 cells were seeded into 96-well plates in the same way as above, and treated with 10 ng/mL TGF-β1 for 12 h to induce EMT after the density was appropriate. A549 cells and HFL-1 cells induced by TGF-β1 were treated with different suitable concentrations of SIN (125, 250, 500, and 1000 µM) for 12 h, and cell viability was measured by CCK-8 reagent used in the same way as above.

### Wound healing assay

A549 cells and HFL-1 cells were seeded into 6-well plates (1 mL/well, NEST, Wuxi, China) at a density of 10^5^ cells per well. They were divided into four groups: control group (without drugs and inducers), TGF-β1 group, TGF-β1 + SIN (500 µM) group and TGF-β1 + SIN (1000 µM) group. Except control group, the A549 and HFL-1 cells induced by TGF-β1 for 12 h were cultured in serum-free F12K medium with or without different concentrations of SIN for 6–12 h. A 200 µL micropipette tip was used to generate horizontal scratches in the center of the well. At different time points, the image of scratch width in each well was obtained by optical microscope imaging and analyzed by ImageJ software. The wound healing rate was calculated as follows = (0 h wound width – 6 h/12 h wound width) / 0 h wound width.

### Detection of cytokines in bronchoalveolar lavage fluid (BALF) and serum of mice

Right lungs were collected and fixed in formalin for 48 h before being processed to make histological slides. Left lungs were flash-frozen in liquid nitrogen for molecular analysis. The washing fluid was collected as the bronchial alveolar lavage fluid (BALF) by using intratracheal perfusion. Blood (1 mL) was collected from the inferior vena cava using a 1 mL syringe. TBALF and serum were placed into a 100 µL tube and stored at -80 ℃. ELISA kits (EK0527, EK0411, EK0398, EK0515, Wuhan Boster Biological Technology, Ltd., Wuhan, China) were used for testing IL-2, IL-6, TNF-α and TGF-β1 level according to the manufacturer’s instructions. The unit of the calculation result was unified as pg/mL.

### Histology and immunohistochemistry

The left lung of the mice was fixed in 4% paraformaldehyde for 2 days, embedded in paraffin, and cut into 4 μm-thick slides. After dewaxing and gradient ethanol hydration, the paraffin sections were stained with hematoxylin-eosin (H&E) staining kit or Masson trichrome staining kit. Three slices were randomly selected from each group, and five views were randomly selected from each slice under the microscope at 200x magnification. Assessment of outcome was performed by pathologists blinded to the treatment group, and the degree of lung injury was scored according to a method previously reported [22]. In addition, the Ashcroft score [23] was used to evaluate the PF. As regards immunohistochemistry, 3% hydrogen peroxide solution was added dropwise to tissue sections to quench endogenous peroxidase activity. Then, the slices were immersed in citrate antigen repair solution (AR0024, Wuhan Boston Biological Technology, Ltd., Wuhan, China), and the antigen was repaired by heat-induced epitope repair method. The follow-up steps were performed in accordance with the manufacturer’s instructions. The freshly prepared DAB working solution (AR1022, Wuhan Boston Biological Technology, Ltd., Wuhan, China) was dropped on the glass slide and the slices were counterstained with hematoxylin for 2 min. The stained tissue sections were observed under a microscope (Leica, DM3000) and the staining degree was evaluated. The antibodies used are listed in supplementary material, Table S1.

### Determination of hydroxyproline content

The right lung was accurately weighed according to the instructions of the hydroxyproline test kit (Nanjing Jiancheng Bioengineering Institute, Nanjing, China). The results were expressed as µg of hydroxyproline/mg of protein.

### Western blot analysis

Cell and mouse lung were lysed using RIPA buffer (P0013, Beyotime, Shanghai, China) containing PMSF (ST506, Beyotime, Shanghai, China), protease inhibitor, and phosphatase inhibitor (P1050, Beyotime, Shanghai, China), and centrifuged at 12,000 rpm and 4 ℃. The concentration of total proteins was detected by BCA kit (P0009, Beyotime, Shanghai, China). The protein samples were diluted with 5× sodium dodecyl sulfate-polyacrylamide gel electrophoresis (SDS-PAGE, P0015, Beyotime, Shanghai, China) and boiled for 5 min. An amount of 30 µg of the total proteins was added to each lane of the SDS-polyacrylamide gel, then they were transferred to a PVDF membrane (Immobilon-P, Darmstadt, Germany) and incubated with the primary antibody at 4 ℃ overnight, followed by incubation with HRP-labeled secondary antibody for 1 h at room temperature. An ECL chemiluminescence detection kit (P00185, Beyotime, Shanghai, China) was used for visualization and imaging with Tanon 5200 automatic chemiluminescence fluorescence image analysis system (Shanghai, China). The intensity of the bands was analyzed and standardized by the internal control (GAPDH) of each sample using ImageJ (Version 1.37c, Bethesda, MD, USA). The antibodies used are listed in supplementary material, Table S2.

### RNA isolation and quantitative real-time PCR (qRT-PCR)

At the end of treatment, total RNA was isolated from the mouse lung and HFL-1 cells by RNA-Quick Purification Kit (ES Science Biotechnology, Shanghai, China) according to the manufacturer’s instructions. GAPDH was used as the internal control for HFL-1 cells and mouse lung. RNA amplification kit SYBR® Premix EX TAG™ ІІ (Takara Biotechnology Co., Ltd., Dalian, China) was used and qRT-PCR was performed using LightCycler® 480 II system (Roche, Alameda, CA, USA). The relative quantification of mRNA expression was calculated according to the 2^−∆∆Ct^ method, where Ct is the cycle threshold. Primers were synthesized by Shanghai Sangon Bioengineering Co., Ltd. The primers used are listed in Table S3.

### 10. Immunofluorescence by confocal microscope

Cells were seeded in confocal Petri dishes (#801,001, NEST, Wuxi, China) and incubated overnight. After EMT induction and SIN treatment, 4% paraformaldehyde (PFA, P0099, Beyotime, Shanghai, China) was added at room temperature for 15 min. Hydrogen peroxide 3% was used to block endogenous peroxidase activity. Cells were treated with Triton X-100 (P0096, Beyotime, Shanghai, China) for 15 min. Then, they were incubated with QuickBlockTM immunostaining blocking solution (P0260, Beyotime, Shanghai, China) for 15 min at room temperature to block nonspecific binding. Afterward, the primary antibody was added and incubated at 4 ℃ overnight, and the appropriate HRP-labeled secondary antibody was added and incubated at room temperature for 1 h. The nucleus was stained with DAPI solution (P0131, Beyotime, Shanghai, China), incubated for 15 min and the slides were sealed. The glass slide was observed under a confocal microscope (Leica Stellaris) and imaged. The antibodies used are listed in supplementary material, Table S4.

###  Statistical analysis

Statistical analysis was performed using GraphPad Prism (version 6.01). Student *t-*test was used for the comparison between two groups, and one-way analysis of variance was used for the comparison among more than two groups. Results are expressed as mean ± SEM of at least three independent experiments (*n* ≥ 3). A value of *p* < 0.05 was considered statistically significant.

## Results

### SIN reduces BLM-induced PF in mice

**Fig. 1:**
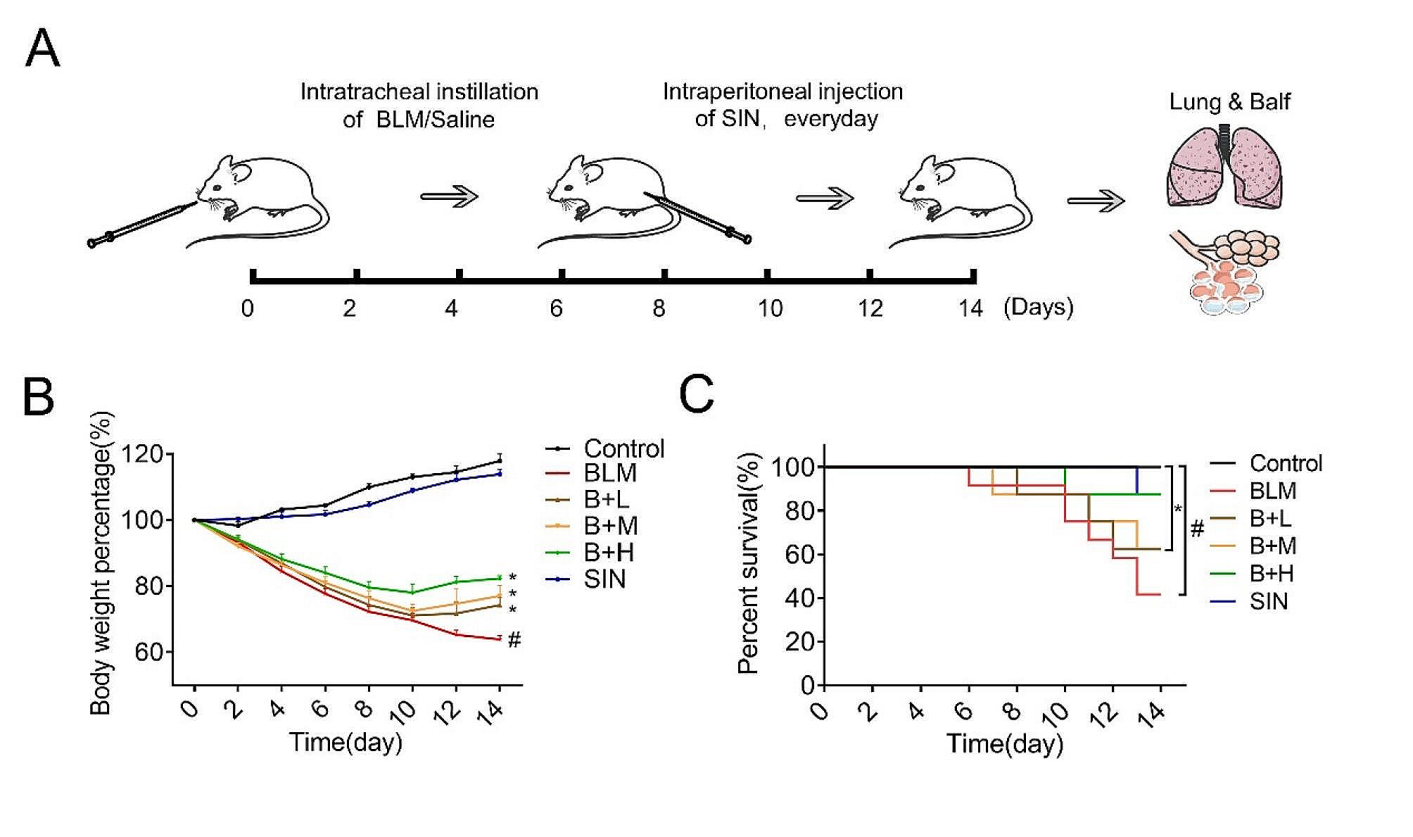
SIN reduces BLM-induced PF in mice. (**A**) C57BL-6 J mice were treated with BLM or 0.9% NaCl solution as BLM (control) at a dose of 3 mg/kg; BLM-induced mice were treated with an intraperitoneal injection of SIN (50, 100 and 150 mg/kg) or vehicle once a day for 2 weeks from the first day after intratracheal instillation of the drug (vehicle). Lungs were collected at designated time points for H&E staining, Masson staining, BALF, hydroxyproline, and other indicators. (**B, C**) Body weight of mice monitored every day during the treatment, and assessment of the survival rate in each group. *n* = 6 mice per group. # *p* < 0.05, compared with control; * *p* < 0.05, compared with BLM; one-way analysis of variance

A preclinical IPF model was established by BLM to evaluate the potential anti-fibrosis effect of SIN in vivo (Fig. 1A). Mice treated with BLM began to die on day 6. The weight loss trend in the SIN treatment group was less and the weight increased with the increase of the treatment dose at a certain range as compared with the BLM group (Fig. 1B, C), although the dose of 200 mg/kg SIN also showed certain toxic effects (**Fig. ****S1****A, B**). The mortality of the BLM group on day 10 and 14 was 33–69%. In contrast, the mortality of mice treated with SIN was 12.5–37.5% (Fig. 1C). In addition, the weight of the drug control group increased rapidly after one week compared with the model group, and the weight gain reached that of the control group.

### SIN attenuates lung tissue injury and fibrosis in a dose-dependent manner

**Fig. 2:**
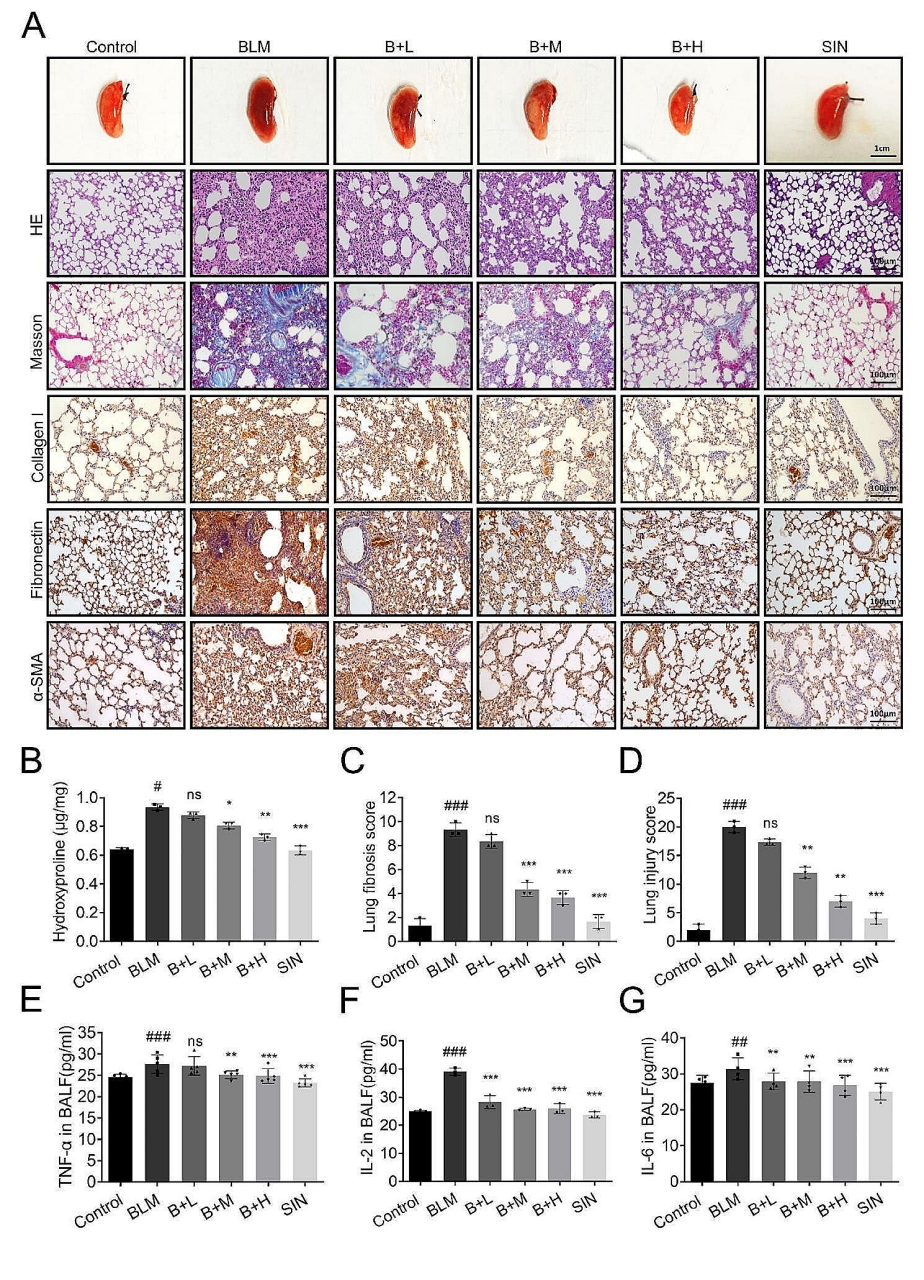
SIN attenuates lung tissue injury and fibrosis in a dose-dependent manner. (**A**) Morphological changes of mouse lung: pink represents normal lung tissue and dark red represents fibrotic lung tissue. Representative micrographs (200x magnification) of paraffin-embedded lung tissue sections stained with H&E and Masson trichrome. Effect of SIN on the expression of collagen I, fibronectin, and a-SMA proteins in the lung of mice after BLM-induced PF on day 14 (200x magnification). (**B**) Analysis and quantification of the content of hydroxyproline in the lung of different groups. (**C**) PF score. (**D**) Lung injury severity score. (**E-G**) Concentrations of TNF-α, IL-2, and IL-6 in BALF detected by ELISA kit. *n* = 6 mice per group. Scale bar: 100 μm. # *p* < 0.05, ## *p* < 0.01, ### *p* < 0.001, compared with control; * *p* < 0.05, ** *p* < 0.01, *** *p* < 0.001, and ns, not significant, compared with BLM; one-way analysis of variance

The lungs of the PF model mice are dark red and lack the spongy texture, while the control mice showed pink and soft lungs. Surprisingly, SIN treatment improved the color and texture of the lungs (Fig. 2A). H&E staining showed pathological changes in the lungs, and the normal alveolar structure in the BLM group was blurred or disappeared, with incomplete alveolar shape, diffuse fibrosis, and inflammatory cell infiltration. In addition, SIN helped the lung to keep intact most of the alveolar structure and significantly reduced the fibrotic lesions. The higher the dose, the less proliferating cells (Fig. 2A). The inflammation of the lungs in the SIN plus BLM group was gradually relieved and the degree of relief was dose-dependent, although edema, inflammatory cell infiltration, and vascularization still existed in some areas, as compared with the control group (Fig. 2A). Masson staining showed that the lung in the control group had a small amount of blue staining, which represented the collagen component of the ECM of the normal lung. After 14 days, a large amount of blue staining appeared in the lung of the BLM group mice, suggesting the deposit of a large amount of collagen due to inflammation. With the increase of the SIN dose, the blue staining gradually decreased after treatment. Sirius red staining also showed the same trend as Masson (**Fig. ****S1****C**). In addition, the changes in the expression of pulmonary fibrosis marker proteins such as a-SMA, collagen I, fibronectin, and connective tissue growth factor are important features of pulmonary fibrosis progress. The immunohistochemical detection of pulmonary fibrosis markers in mice showed that a-SMA, collagen I, fibronectin, and connective tissue growth factor in the lung were significantly increased by a single intratracheal administration of BLM, while the concentration of SIN in the treatment group was negatively correlated with the staining degree of fibronectin. In addition, the intraperitoneal injection of SIN did not affect the expression of a-SMA, collagen I, and fibronectin (brown) (Fig. 2A).

Hydroxyproline content indirectly quantifies collagen deposition, since this amino acid exists almost exclusively in collagen. The collagen deposition in the fibrosis focus of BLM mice increased significantly compared with that in the control mice. The continuous treatment with SIN resulted in a gradual reduction of the collagen deposition. It is worth noting that 150 mg/kg of SIN significantly inhibited the collagen deposition (Fig. 2B). The Ashcroft score and lung injury score also showed that lung inflammation and collagen deposition in the BLM group were consistent with the previous trend (Fig. 2C, D). In addition, the degree of inflammation in the lung assessed by the detection of the expression of the inflammatory factors TNF-α, IL-2, and IL-6 in the BALF revealed that BLM caused their significant increase compared with the control group. However, the release of cytokines was inhibited by SIN (Fig. 2E-G).

### SIN alleviates BLM-induced PF, as well as neutralizes ECM deposition and EMT

**Fig. 3:**
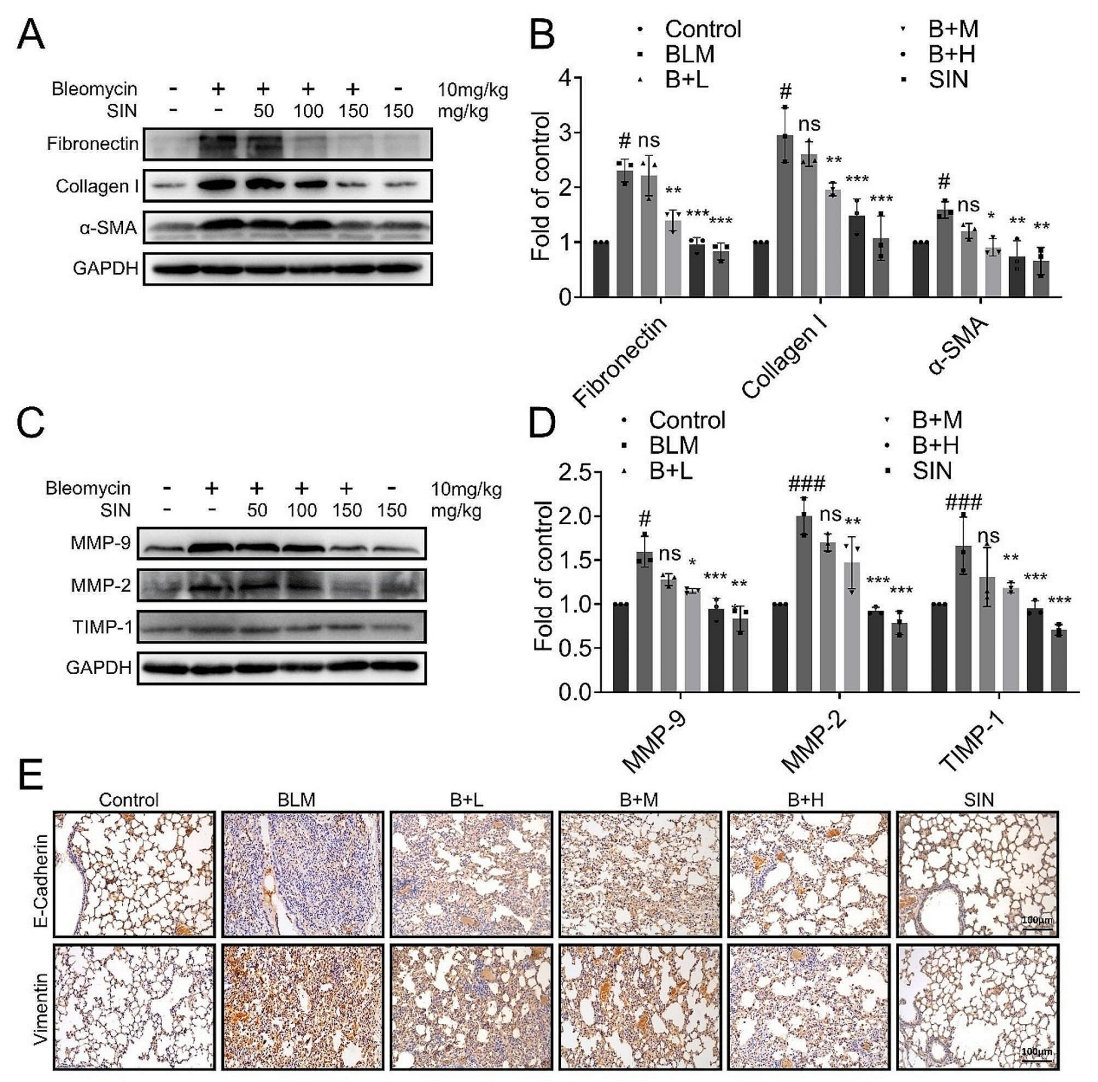
SIN alleviates BLM-induced PF, as well as neutralizes ECM deposition and EMT. (**A**) Western blot image showing the expression of collagen I, fibronectin, and a-SMA in the lung. (**B**) The expression of collagen I, fibronectin, and a-SMA in Figure A was normalized to the expression of GAPDH (*n* = 3). (**C**) Western blot image showing the expression of MMP-9, MMP-2, and TIMP-1 in the lung. GAPDH was used as the loading control. (**D**) The expression of MMP-9, MMP-2, and TIMP-1 in Figure C was normalized to the expression of GAPDH (*n* = 3). (**E**) Effect of SIN on the expression of E-cadherin and vimentin in the lung of BLM-induced PF mice on day 14 by immunohistochemistry (200x magnification). *n* = 6 mice per group. Scale bar: 100 μm. # *p* < 0.05, ### *p* < 0.001, compared with control; * *p* < 0.05, ** *p* < 0.01, *** *p* < 0.001, and ns, not significant, compared with BLM; one-way analysis of variance

Western blot results showed that the expressions of fibronectin, collagen I, and α-SMA in the BLM group were significantly higher than those in the control group, and the expressions of these proteins were effectively inhibited after SIN treatment (Fig. 3A, B). Western blot also showed that the expressions of MMP-9, MMP-2, and TIMP-1 in the lungs of mice in the BLM group were significantly increased, and this trend was effectively suppressed by SIN (Fig. 3C, D).

The classical markers of EMT such as E-cadherin and vimentin, were assessed by immunohistochemistry to verify whether SIN was able to alleviate pulmonary fibrosis by inhibiting BLM-induced EMT [24]. Immunohistochemistry (Fig. 3E) showed the same trend. The expression of vimentin in the lung significantly increased after BLM induction, while the expression of E-cadherin significantly decreased compared with the control group. However, SIN induces a decrease in the expression of vimentin and an increase in the expression of E-cadherin.

### SIN regulates TGF-β1/Smad3 signaling pathway

**Fig. 4:**
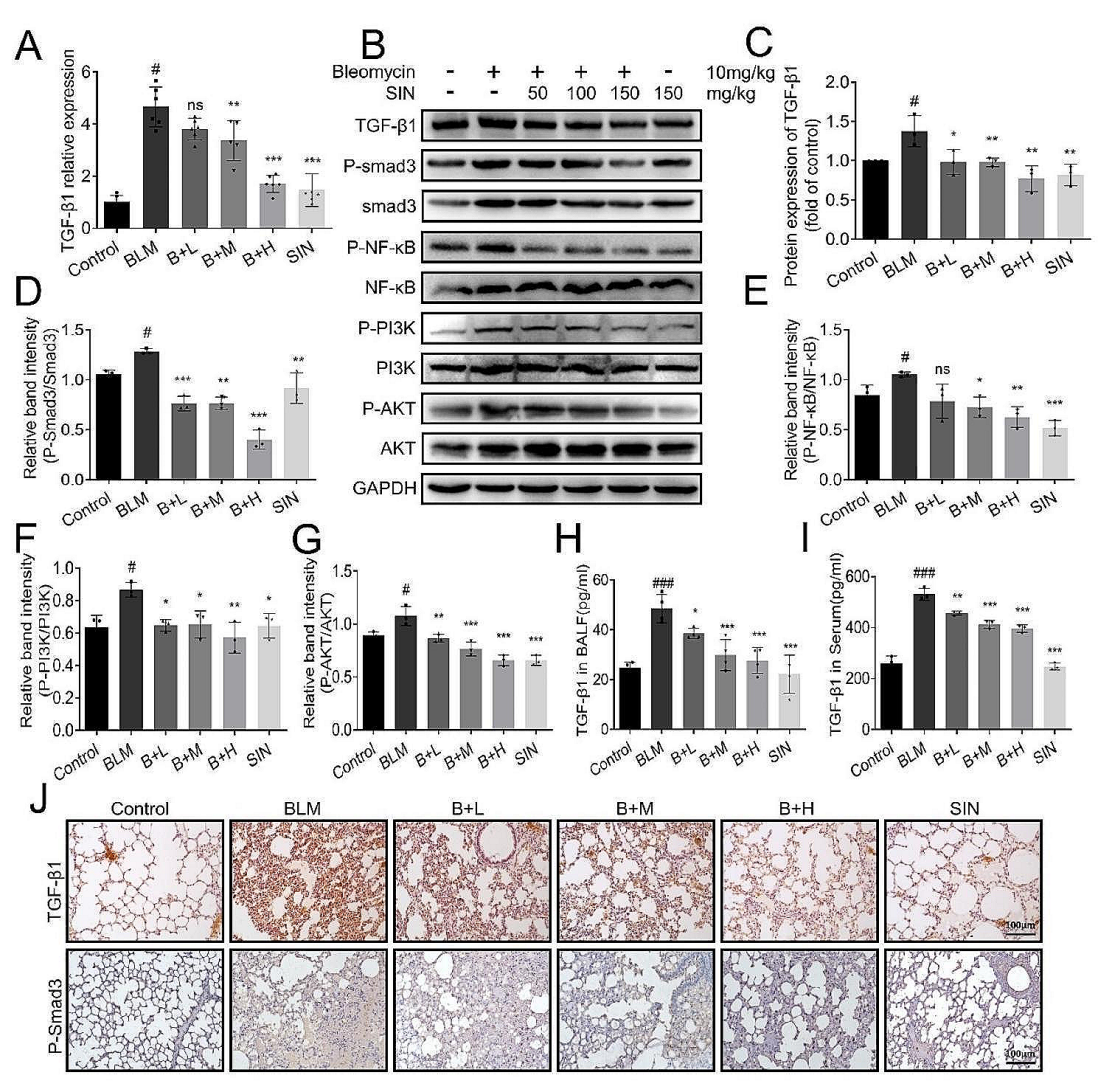
SIN regulates TGF-β1/Smad3 signaling pathway. (**A**) Relative expression of TGF-β1 mRNA in the lung of mice from each group measured by qRT-PCR. (**B**) Western blot image showing the expression trend of P-Smad 3, a protein related to the TGF-β/Smad signaling pathway, and the protein expression trend of the related pathways NF-κB and PI3K/AKT in the lung. (**C-G**) The expression of TGF-β1, P-Smad3/Smad3, P-NF-κB/NF-κB, P-PI3K/PI3K, and P-AKT/AKT was normalized in Figure B (*n* = 3). (**H**) The concentration of TGF-β1 in BALF. (**I**) The concentration of TGF-β1 in serum. (**J**) Effect of SIN on the expression of TGF-β1 and P-Smad3 in the lung of BLM-induced PF mice on day 14 by immunohistochemistry (200x magnification). *n* = 6 mice per group. Scale bar: 100 μm. # *p* < 0.05, ### *p* < 0.001, compared with control; * *p* < 0.05, ** *p* < 0.01, *** *p* < 0.001, and ns, not significant, compared with BLM; one-way analysis of variance

The quantitative analysis of TGF-β1 by qRT-PCR showed that the transcription of TGF-β1 in the BLM group was significantly increased, and it decreased after the treatment with SIN in a dose-dependent manner (Fig. 4A). In addition, the results of the assessment of TGF-β1 signaling pathway revealed that the expression of TGF-β1 in the lung was significantly increased after BLM induction, and the downstream key protein P-smad3 was significantly activated. SIN induced an inhibition in the expression of TGF-β1 and the hyperphosphorylation of Smad3 induced by BLM (Fig. 4B-D). Further, a down-regulatory effect of SIN on the levels of NF-κb and PI3K/AKT signaling pathway-related proteins was detected in BLM-induced mouse lungs (Fig. 4B, E-G). In addition, the results of BALF and serum of mice showed that the up-regulation of serum TGF-β1 stimulated by BLM was significantly inhibited by SIN treatment (Fig. 4H, I). Similarly, the immunohistochemical analysis of the lung showed that TGF-β1 and P-Smad3 had the same trend in SIN-treated mouse lungs (Fig. 4J).

### SIN inhibits the proliferation and migration of HFL-1 and A549 cells induced by TGF-β1

SIN showed an anti-fibrotic effect in the in vivo PF model. Thus, in vitro experiments using two cell lines were performed to further explore the anti-fibrotic mechanism. Epithelial cells have the ability to respond to changes in the microenvironment through EMT. EMT is also the driving force of normal repair, promoting the emergence of myofibroblasts that secrete collagen in IPF. Therefore, the down-regulation of epithelial markers, up-regulation of mesenchymal markers, and ECM deposition are observed during EMT [24].

**Fig. 5:**
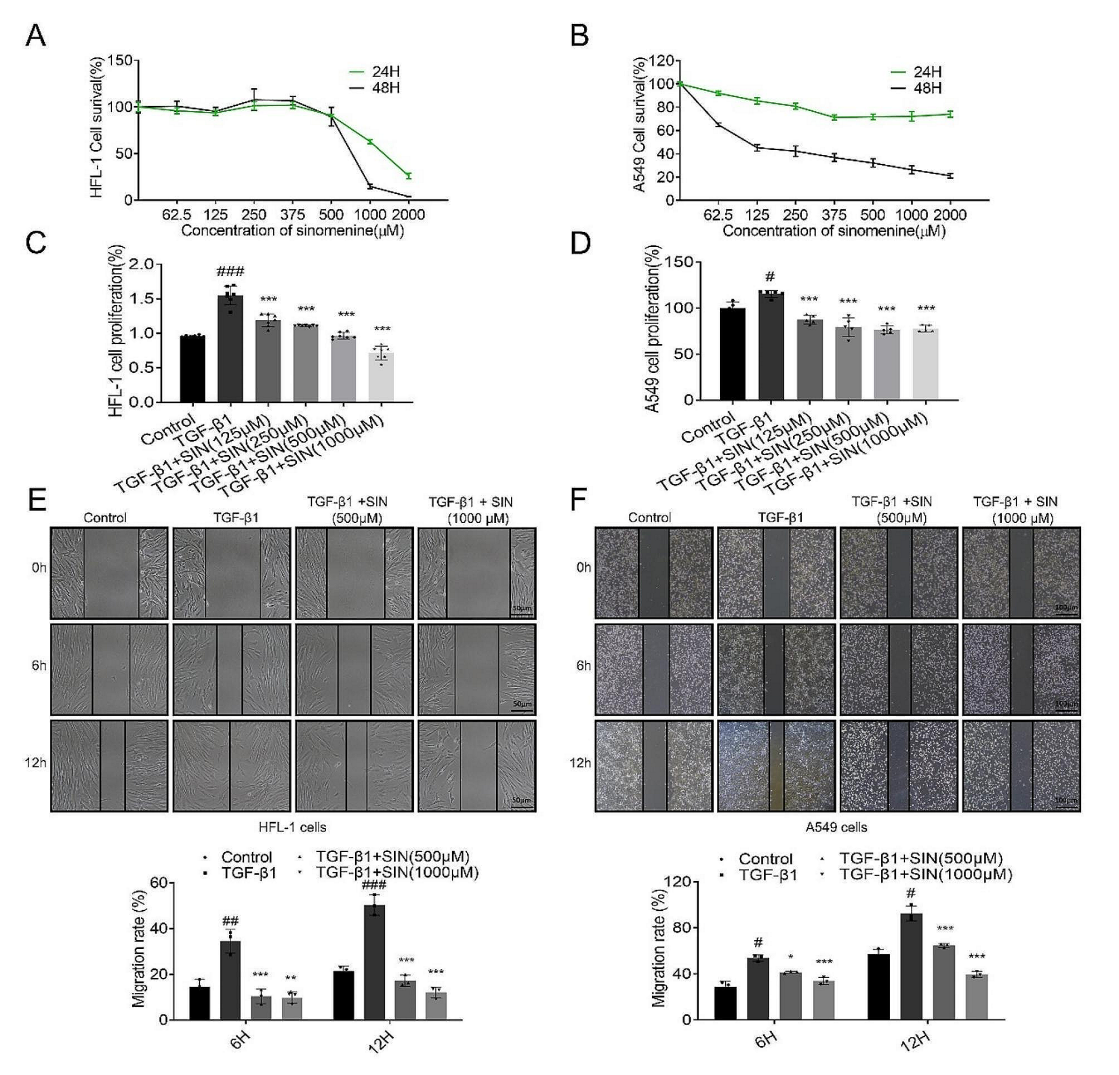
SIN inhibits the proliferation and migration of HFL-1 and A549 cells induced by TGF-β1. (**A, B**) Toxicity of different concentrations of SIN on HFL-1 and A549 cells by CCK-8. (**C, D**) Effect of different concentrations of SIN (125, 250, 500, and 1000 µM) on the proliferation of different cells after TGF-β1 treatment. (**E**) Migration and migration width of HFL-1 cells treated with SIN (500 and 1000 µM) quantified at 0 h, 6 h, and 12 h after TGF-β1 induction. Scale bar: 50 μm. (**F**) Migration and migration width of A549 cells treated with SIN (500 and 1000 µM) quantified at 0 h, 6 h, and 12 h after TGF-β1 induction. Scale bar: 100 μm. *n* = 3. Results are expressed as mean ± SED. # *p* < 0.05, ## *p* < 0.01, ### *p* < 0.001, compared with control. * *p* < 0.05, *** *p* < 0.001, compared with TGF-β1. One-way analysis of variance

In this study, HFL-1 and A549 cells were treated with TGF-β1 to induce EMT in vitro. HFL-1 and A549 cells were treated with SIN at different concentrations, and the results revealed that 62.5–1000 µM SIN did not induce evident cell death within 24 h (Fig. 5A, B). Therefore, the dose of 125–1000 µM was selected for all cell experiments. The proliferation of HFL-1 and A549 cells was promoted when treated with TGF-β1, but the proliferation of HFL-1 and A549 cells was inhibited by TGF-β1 combined with SIN in a dose-dependent manner (Fig. 5C, D). The results showed that the migration of HFL-1 and A549 cells treated with TGF-β1 was increased, but it was inhibited by the treatment of SIN combined with TGF-β1, with a migration inhibition effect that increased in a dose-dependent manner (Fig. 5E **and F**).

### SIN prevents TGF-β1-induced fibroblasts transformation into FMT and improves ECM

**Fig. 6:**
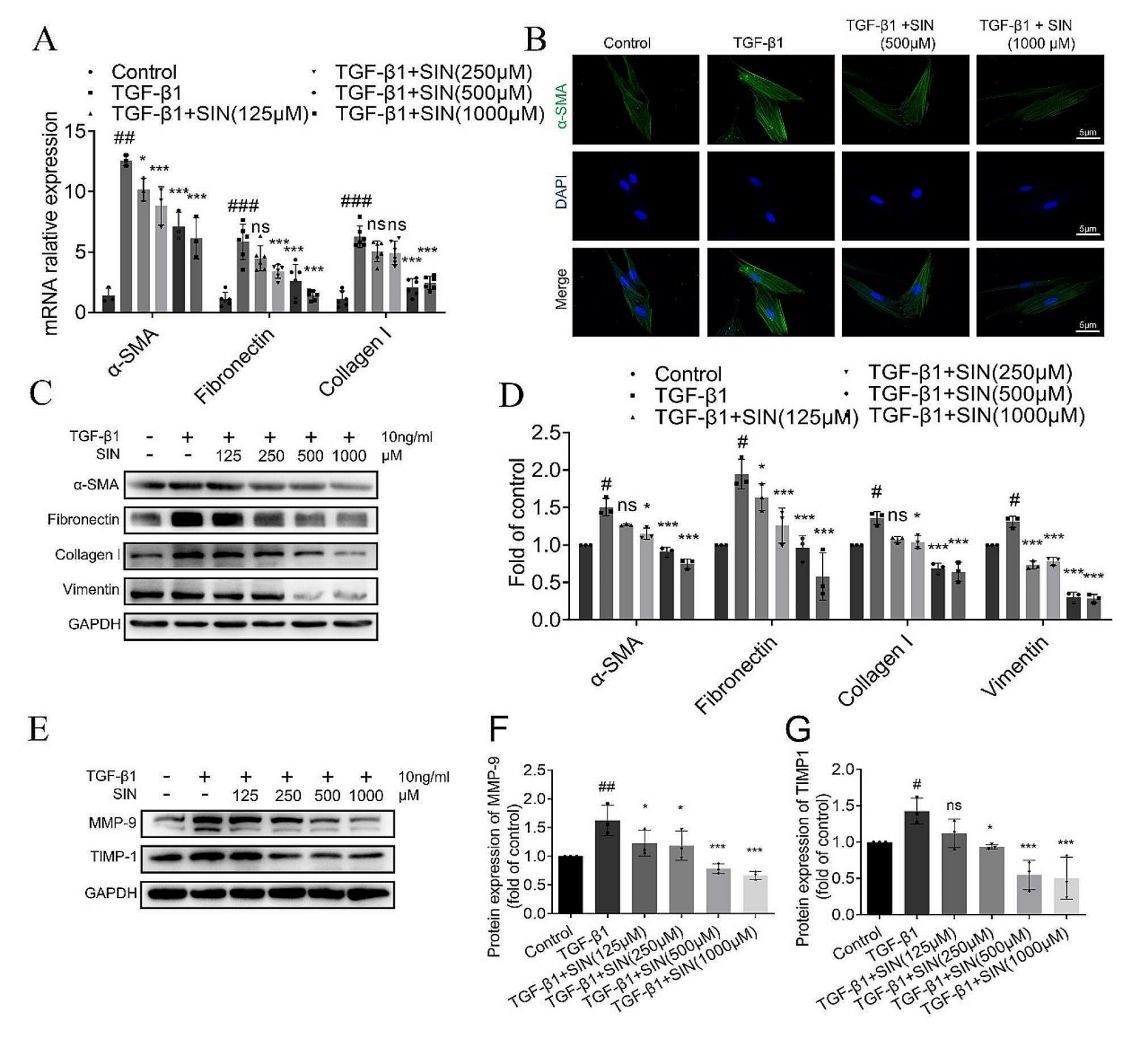
SIN prevents TGF-β1-induced fibroblasts transformation into FMT and improves ECM. (**A**) mRNA expression of α-SMA, fibronectin, and collagen I detected by qRT-PCR. (**B**) HFL-1 cells treated with TGF-β1 were then treated with different concentrations of SIN (500 and 1000 µM), and the representative image of α-SMA protein by immunofluorescence. α-SMA: green; DAPI: blue staining in the nuclei. (**C**) Expression of α-SMA, collagen I, vimentin, and fibronectin in HFL1 cells treated with SIN by western blot (125, 250, 500, and 1000 µM). (**D**) The expression of different proteins in Figure C was normalized to GAPDH expression. (**E**) Expression of MMP-9 and TIMP1 by western blot. (**F, G**) The expression of different proteins in Figure E was normalized to GAPDH expression. *n* = 3. Results are shown as mean ± SEM. Statistical analysis was performed by one-way analysis of variance. Scale bar: 5 μm. # *p* < 0.05, ## *p* < 0.01, ### *p* < 0.001, compared with control. * *p* < 0.05, *** *p* < 0.001 and ns, not significant, compared with TGF-β1

The mRNA expression of α-SMA, collagen I, and fibronectin significantly increased in the TGF-β1 group, and decreased after the treatment with SIN in a dose-dependence manner (Fig. 6A). The protein expression of α-SMA in HFL-1 cells increased after TGF-β1 treatment, but it was inhibited after SIN treatment (Fig. 6B). In addition, the expression of a-SMA, collagen I, vimentin, and fibronectin in the TGF-β1 group was significantly higher than that in the control group, and the expression of the above proteins was effectively inhibited by SIN (Fig. 6C, D). The protein expression of MMP-9 and TIMP-1 in HFL-1 cells induced by TGF-β1 was significantly increased compared with that in the control group, but their expression was down-regulated by SIN (Fig. 6E-G).

### SIN reverses ECM and EMT induced by TGF-β1 in A549 cells

**Fig. 7:**
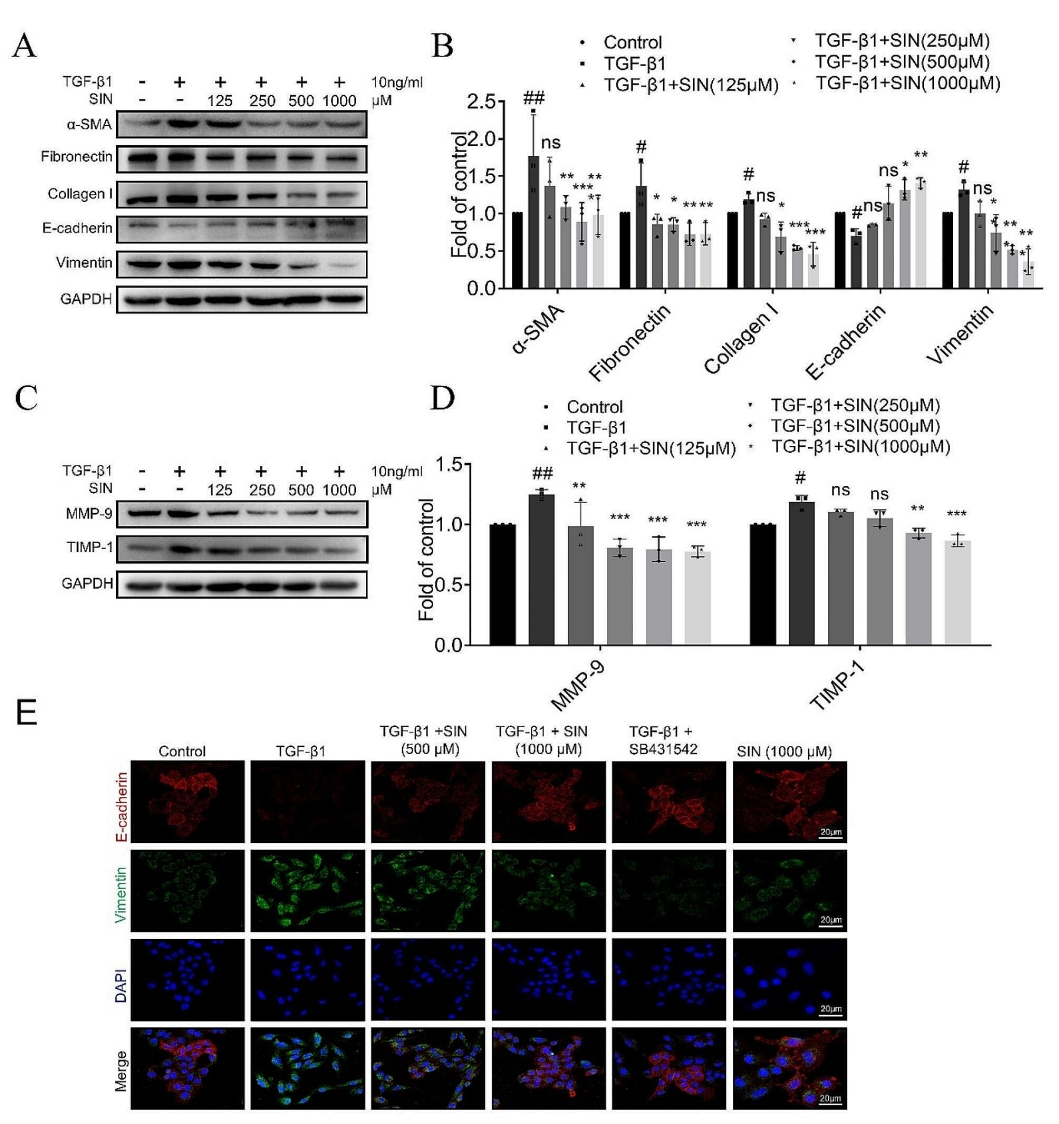
SIN reverses ECM and EMT induced by TGF-β1 in A549 cells. (**A**) Expression of α-SMA, fibronectin, collagen I, E-cadherin, and vimentin in A549 cells treated with SIN (125, 250, 500, and 1000 µM) by western blot. (**B**) The expression of different proteins in Figure A was normalized to GAPDH expression. (**C**) Expression of MMP-9 and TIMP1 in A549 cells by western blot. (**D**) The expression of different proteins in Figure C was normalized to GAPDH expression. (**E**) A549 cells were treated with SB431542 as control, and representative images of E-cadherin and vimentin by immunofluorescence in each group. Scale bar: 20 μm. *n* = 3. Results are shown as mean ± SEM. Statistical analysis was performed by one-way analysis of variance. # *p* < 0.05, ## *p* < 0.01, compared with control. * *p* < 0.05, *** *p* < 0.001 and ns, not significant, compared with TGF-β1

The anti-fibrotic effect of SIN on A549 cells was confirmed by the ability of SIN to reduce the expression of α-SMA, fibronectin, and collagen I that were increased by TGF-β1 (Fig. 7A, B). In addition, the protein expression of MMP-9 and TIMP-1 in A549 cells induced by TGF-β1 was significantly increased compared with that in the control group, while their expression was also decreased by SIN (Fig. 7C, D). Moreover, the expression of E-cadherin decreased in TGF-β1 group compared with that in the control group, while the expression of vimentin increased, while the treatment with SIN combined with TGF-β1 significantly increased the expression of E-cadherin and decreased the expression of vimentin (Fig. 7A, B, E). Finally, the trend after the treatment with SB-431,542 (10 mM) combined with TGF-β1 was basically the same as that after the treatment with SIN combined with TGF-β1 (Fig. 7E).

### Effect of SIN on TGF-β1/Smad3, PI3K/Akt and NF-κB pathways in HFL-1 cells induced by TGF-β1

**Fig. 8:**
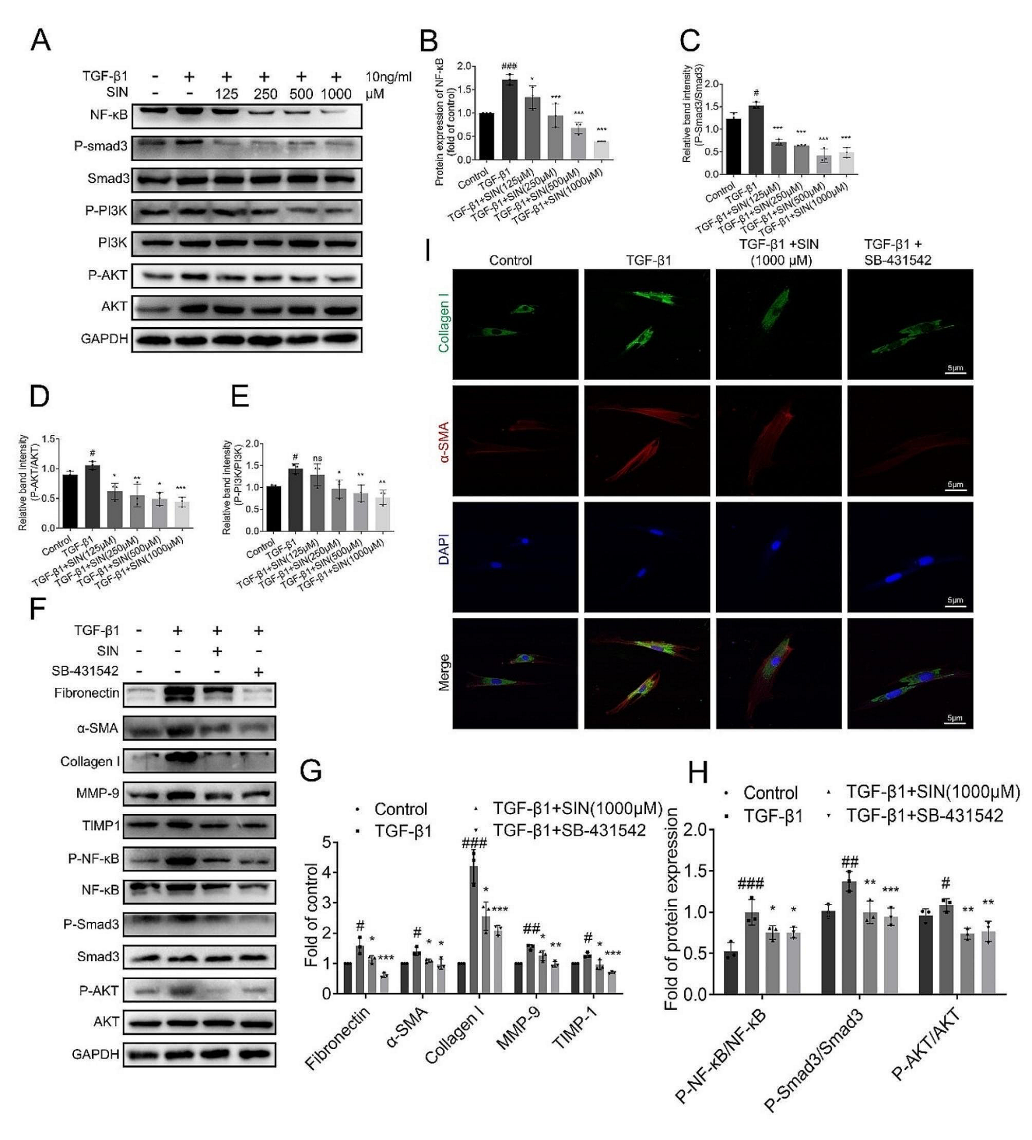
Effect of SIN on TGF-β1/Smad3, PI3K/AKT and NF-κB pathways in HFL-1 cells induced by TGF-β1. (**A**) Expression of NF-κB, Smad3, and PI3K/AKT signaling pathway analyzed by western blot. (**B-E**) The expression of the different proteins in Figure A was normalized to GAPDH expression (*n* = 3). (**F**) Expression of fibronectin, α-SMA, collagen I, MMP-9, and TIMP-1 in HFL-1 cells analyzed by western blot in the following four groups. Control group treated with nothing, TGF-β1 group treated with TGF-β1 (10 ng/ml) for 12 h, the treatment group was treated with SIN (1000 µM) for 12 h after TGF-β1, and the TGF-β receptor inhibitor group was not treated with SIN after SB-431,542. (**G, H**) The expressions of different protein in Figure F are normalized (*n* = 3). (**I**) Representative images showing the co-localization of collagen I (green) and α-SMA (red), and the nuclei stained by DAPI (blue). *n* = 3. Scale bar: 5 μm. Results are shown as mean ± SEM. Statistical analysis was performed by one-way analysis of variance. # *p* < 0.05, ## *p* < 0.01, ### *p* < 0.001, compared with control. * *p* < 0.05, ** *p* < 0.01, *** *p* < 0.001, compared with TGF-β1

The phosphorylation level of Smad3, PI3K/Akt, and the protein expression of NF-κB in HFL-1 cells treated with TGF-β1 alone or in combination with SIN were evaluated to explore the regulatory effect of SIN on the signaling pathway in the HFL-1 cells. The results showed that SIN decreased the increase of phosphorylated Smad3, PI3K/Akt, and NF-κB in TGF-β1-induced cells without changing the overall levels of Smad3 and PI3K/Akt (Fig. 8A-E). These results suggested that SIN inhibited TGF-β1-induced fibrosis in HFL-1 cells by inhibiting Smad3, PI3K/Akt, and NF-κB signaling pathways.

3.9. **SIN plays an anti-fibrotic role by regulating TGF-β1-induced abnormal activation of Smad pathway and non-Smad downstream pathway.**

The treatment with SIN (1000 µM) for 12 h significantly reduced the protein expression of a-SMA, collagen I, and fibronectin induced by TGF-β1, and the degree of inhibition was similar to that induced by SB-431,542 combined with TGF-β1 (Fig. 8F, G). Furthermore, SIN combined with TGF-β1 inhibited the expression of MMP-9 and TIMP-1 to some extent compared with TGF-β1, and the degree of inhibition was similar to that of SB-431,542 combined with TGF-β1 (Fig. 8F, G). In addition, the expression of collagen I and α-SMA in HFL-1 cells induced by TGF-β1 showed that SIN reversed the fibrosis markers induced by TGF-β1, which was consistent with the above trend (Fig. 8I). Finally, the expression of the core functional proteins P-NF-κB、P-Smad3 and P-AKT of the above-mentioned pathways were evaluated and the results revealed that SIN decreased the expression of the proteins mentioned above by different degrees, which was similar to the trend obtained by the treatment with SB-431,542 combined with TGF-β1 treatment group (Fig. 8F, H, S1E, S1F).

## Discussion

IPF is a progressive disease, with most of the patients worsening within 2–3 years after diagnosis, with a mortality rate of 50% [25]. The outbreak of COVID-19 resulted in the occurrence of complications including IPF in critically ill patients, making it one of the main problems that need to be solved and effectively treated [26]. IPF is a progressive disease caused by the continuous injury of the alveolar epithelium, which leads to the continuous activation of repair mechanisms, the uncontrolled proliferation and differentiation of fibroblasts into myofibroblasts, and then excessive proliferation, EMT and ECM production as well as collagen deposition in the affected organs [27]. In the initial stage of IPF, a variety of proinflammatory factors (such as TNF-α, IL-6, IL-1, and TGF-β) and matrix metalloenzymes secreted by fibroblasts participate in the chemotaxis and proliferation of inflammatory cells and the mediation of intercellular interaction, promoting the further progress of inflammatory repair reaction [28]. NF-κB is one of the classical molecules involved in inflammation, usually activated to protect it from pathogens, but its abnormal activation is usually the cause of chronic inflammation.

SIN has anti-rheumatic and pharmacological effects, as demonstrated already in the 70s [29]. At present, SIN has been approved by China Food and Drug Administration (CFDA) for the treatment of rheumatoid arthritis (RA), but its mechanism of action against other diseases has not been fully clarified. Previous studies reported that SIN has anti-inflammatory effects in adjuvant arthritis rats, and it regulates T cells and Th17 cells in intestinal-associated lymphoid tissues [28, 30]. Similarly, studies have shown that Sinomenine hydrochloride can treat NSCLC and RA by the specific mechanism of selectively inhibiting the growth of NSCLC cells and the progression of RA through activation of the AMPK pathway, providing new insights into the treatment of tumors and autoimmune diseases [31]. Based on the current situation, there is no doubt that SIN possesses anti-tumor and anti-fibrosis ability. The fact is that in clinical practice the incidence of concurrent lung cancer in patients with interstitial lung abnormalities or pulmonary fibrosis increases despite cumulative increases in survival time, but a worldwide questionnaire showed great heterogeneity in the strategy and quality of management of IPF-lung cancer [32]. The PD-1/PD-L1 pathway is currently one of the main targets of immunologic drugs for the treatment of lung cancer, and some studies have shown that IPF patients with concurrent enlarged lymph nodes have higher PD-1 expression and CD4/CD8 ratios, whereas pembrolizumab shows antifibrotic effects in the bleomycin model in mice [33, 34]. In addition, the dual-specificity protein phosphatase protein family (DUSP10/MKP-5, DUSP9/ MKP-4) shows antagonistic effects in cancer and fibrosis [35, 36]. This reminds us that for this easily overlooked population, they urgently need to find a common target of anti-cancer and anti-fibrosis, and also need a consensus statement of unified standard methods. The present study showed that the antifibrotic effect of SIN was associated with the regulation of TGF-β1/Smad3, PI3K/Akt and NF-κB signaling pathways.

Fibroblasts are vital in the formation of IPF structure and in the maintenance of the lung tissue function. The continuous proliferation of fibroblasts combined with alveolar epithelial cells interaction due to the stimulation of cytokines promotes fibrosis [37, 38]. In the process of alveolar epithelial damage and abnormal repair, excessive collagen deposition occurs in the tissue, and fibroblasts are transformed into myofibroblasts, with α-SMA being the main sign of the transformation from fibroblasts to myofibroblasts [39]. Our results showed that SIN inhibited the proliferation and migration of HFL-1 cells in a dose-dependent manner. MMPs are a group of enzymes mainly responsible for the degradation of ECM, but their activity increases during the pathogenesis of PF and the repair or remodeling of inflammatory tissues [37, 40]. TIMPs, which inhibit MMP cleavage activity, are abundant in lung parenchyma of IPF patients and animals with induced PF, which contributes to the failure of degradation of collagen and other ECM components [41]. This study showed that SIN inhibited the abnormal expression of TIMP-1 and MMP-2/9 caused by pulmonary fibrosis and improved the physiological homeostasis of ECM. Therefore, our hypothesis was that SIN restored the imbalance of MMPs/TIMPs ratio caused by PF, delaying its further development.

EMT is a key step in the process of PF. Lung epithelial cells are common targets of injury, the driving force of normal repair, and the key factor of fibrotic lung disease. An important feature of epithelial cells is that they have the ability to respond to microenvironment signals through EMT. EMT regulation consists of a series of key steps to produce pro-inflammatory signals that cause cell damage. EMT is not the transformation from alveolar epithelial cells to fibroblasts, but the ability to reversibly acquire mesenchymal characteristics and enhance mesenchymal crosstalk [8, 42]. In these highly regulated repair pathways, repeated injuries are superimposed with persistent inflammation and hypoxia, which leads to excessive ECM deposition in activated fibroblasts, which in turn destroys normal lung structure and affects gas exchange [9]. Once this positive feedback mechanism is formed, PF continues to progress [43]. Our experiments in vivo showed that SIN inhibited the increase of collagen I, fibronectin, and α-SMA protein expression induced by BLM. Furthermore, TNF-α, IL-1β1, IL-6, and TGF-β1 in BALF of mice significantly decreased after SIN treatment, as well as TGF-β1 in the serum. The expression of NF-κB protein also significantly decreased in a dose-dependent manner in the SIN treatment group, suggesting that the potential mechanism to inhibit the inflammatory response might be the down-regulation of NF-κB expression and the inhibition of the downstream release of inflammatory cytokines. Interestingly, SIN reversed the expression of E-cadherin and vimentin in A549 cells treated with TGF-β1, and also reduced the expression of vimentin and α-SMA in HFL-1 cells treated with TGF-β1 to inhibit EMT. Our research results showed for the first time that SIN alleviated the damage in the structure of the lung, collagen fiber deposition, and inflammatory cell accumulation induced by BLM, significantly inhibited the release of inflammatory cytokines in the lung, reversed EMT, and improved PF.

Next, the molecular mechanism used by SIN to alleviate PF was investigated. TGF-β/Smad is recognized as a key signaling pathway in the process of fibrosis [44, 45]. Our experiment showed that BLM led to a significant increase in the expression of TGF-β1 and P-Smad3 proteins in the lung, and SIN selectively reduced the accumulation of TGF-β1 and P-Smad3. TGF-β1 induced the proliferation of HFL-1 cells in a time-dependent manner, while SIN significantly reduced the cell proliferation induced by TGF-β1 in a dose-dependent manner. Many studies reported that TGF-β/Smad and PI3K/Akt signaling pathways are involved in regulating the formation of PF [46, 47]. Based on the fact that the increased TGF-β1 production in BLM-induced IPF was effectively inhibited by SIN, the anti-fibrotic effect of SIN might be due to the inhibition of Smad and PI3K/Akt signaling pathways. TGF-β1/Smad3 and PI3K/Akt signaling pathways were activated by TGF-β1, and the expression of phosphorylated proteins in Smad3 and PI3K/Akt signaling pathways was significantly inhibited by SIN. This suggested that SIN blocked the TGF-β1/Smad3 and PI3K/Akt signaling pathways. The effect of SIN on the downstream target of TGF-β1 was confirmed using SB-431,542 to explore its mechanism. Indeed, the expression of fibronectin, α-SMA, type I collagen, and MMP-related proteins in HFL-1 cells pretreated with SB-431,542 was inhibited compared with their expression in the TGF-β1 group, and the same trend was observed in the SIN treatment group, but the degree of inhibition was not complete. NF-κB, P-smad3, and P-AKT were significantly decreased in the inhibitor group, and the slight difference from the treatment group might be due to the interaction and relationship between signaling pathways. The up-regulation of collagen and α-SMA expression in the TGF-β1 group indicated that fibroblasts were transformed into myofibroblasts, while the expression of the same proteins in the SIN and SB-431,542 group was inhibited, indicating that SIN inhibited the differentiation and collagen deposition of HFL-1 cells. These results suggested that SIN also blocked the non-Smad signaling pathways downstream of TGF-β1, including PI3K/AKT and NF-κB pathways, during the treatment of PF.

In this study, SIN modulation of TGF-β1 targeted regulation of NF-κB and PI3K/AKT pathways to achieve remission of pulmonary fibrosis, which is promising to provide ideas for the in vivo typing-targeted treatment of IPF, as well as contributing to the precision medicine treatment of IPF. Based on the fact that precision medicine approaches have revolutionized the clinical management of other diseases such as lung cancer and asthma, the implementation of precision medicine for IPF has become an urgent need for technology and experience in the clinic [48, 49]. Although the two current antifibrotic drugs (pirfenidone and nintedanib) have been shown to slow the progression of the disease, they continue to be administered indiscriminately and are uniformly referred to as “idiopathic pulmonary fibrosis”. In an explosion of studies in the last decade, genetic variants, peripheral blood expression, microRNA and telomere shortening have been shown to predict the risk of progression and death in IPF. There is also an urgent need for biomarkers that can predict and measure the response to IPF treatment, and a relatively large number of relevant indicators have emerged for both drugs after treatment, such as CA-125, serum SP-D, KL-6 and CCL18 [50]. Also the results of the recent PRECISIONS trial are highly anticipated, which aims at addressing whether NAC has a differential effect on the progression of pulmonary fibrosis depending on the TOLLIP gene variant. Therefore, biomarkers associated with disease activity may be closely related to the stratification of patients’ conditions, the determination of the timing of intervention, the categorization of specific molecular types of pulmonary fibrosis, and the designation of future treatment regimens with targeted therapies.

**Fig. 9:**
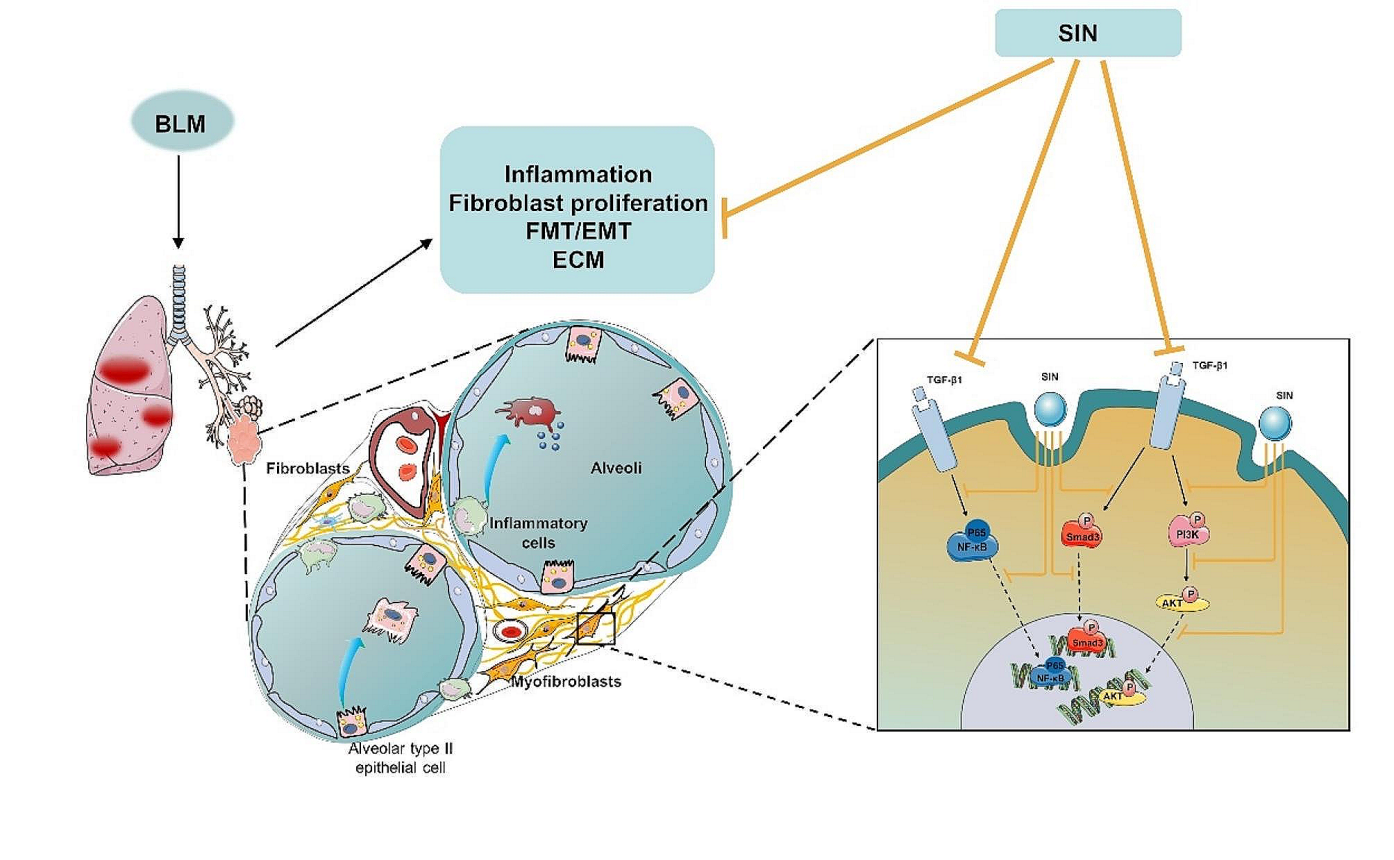
Summary of the effect of SIN on pulmonary fibrosis. SIN exerts anti-fibrotic effects through regulating TGF-β1/Smad3、PI3K/AKT and NF-κB pathways negatively, and inhibiting epithelial-mesenchymal transition and fibroblast proliferation, reducing collagen deposition, and alleviating the inflammatory response

Overall, our results demonstrated that SIN inhibited TGF-β1/Smad3, PI3K/Akt, and NF-κB pathways to produce its therapeutic effects on PF (**Fig. 9**). However, considering the shortcomings of the experimental design and the problems to be solved in the future, further studies should be performed to assess whether SIN treatment on TGF-β1-induced cells mediates other mechanisms, such as the expression of autophagy-related proteins downstream of Akt. It is hoped that this result will help SIN to treat patients with specific genotypes and become a precise medical method.

## Conclusion

SIN is a natural monomer used in traditional Chinese medicine that might become an effective drug for treating PF. Indeed, our evidence revealed that SIN might represent a more effective way in the clinical treatment of the consequences of SARS-COV-2 PF, providing ideas for the treatment of IPF with traditional Chinese medicine.

### Electronic supplementary material

Below is the link to the electronic supplementary material.

Supplementary Material 1

## Data Availability

All data generated or analyzed in this study are included in this article. Other data that are relevant to this article are available from the corresponding author upon reasonable request.

## Abbreviations

H&E: Haemotoxylin and eosin stain
Akt: Protein kinase B
HFL-1: human embryonic lung fibroblasts
ARDS: acute respiratory distress syndrome
IL: interleukin
BLM: bleomycin
IPF: Idiopathic pulmonary fibrosis
BCA: Bicinchoninic acid
NF-κB: nuclear factor κB
BALF: bronchoalveolar lavage fluid
PF: Pulmonary fibrosis
DAB: 3,3’-Diaminobenzidine
PI3K: Phosphoinositide 3-kinase
EMT: epithelial-mesenchymal transition
PMSF: Phenylmethanesulfonyl fluoride
ECM: extracellular matrix
PVDF: polyvinylidene fluoride
ECL: Electrochemiluminescence
SIN: Sinomenine
ELISA: Enzyme-linked immunosorbent assay
SARS-CoV-2: the severe acute respiratory syndrome coronavirus type 2
FMT: transformation of fibroblasts into myofibroblasts
SDS-PAGE: sodium dodecyl sulfate polyacrylamide gel electrophoresis
FBS: fetal bovine serum
TNF-α: tumor necrosis factor α
FDA: Food and Drug Administration
TGF-β1: Transforming growth factor1
