# Supplementary material for: Sinomenine attenuates pulmonary fibrosis by downregulating TGF-β1/Smad3, PI3K/Akt and NF-κB signaling pathways
Source: BMC Pulm Med. 2024 May 10;24:229. doi: 10.1186/s12890-024-03050-5 (PMC11088103; doi:10.1186/s12890-024-03050-5)
Supplement: Supplementary file 1 — Supplementary Material 1 [file 12890_2024_3050_MOESM1_ESM.docx]

**Table S1** Antibodies used for Histology and immunohistochemistry

| Antibody | **Cat No.** | Supplier |
| --- | --- | --- |
| NF-κB | 8242 | Cell Signaling Technology |
| Akt | 9272 | Cell Signaling Technology |
| phospho-Akt | 4060 | Cell Signaling Technology |
| PI3K | WL02849 | WanleiBio |
| phospho-PI3K | 4228 | Cell Signaling Technology |
| E-cadherin | 14472 | Cell Signaling Technology |
| vimentin | 5741 | Cell Signaling Technology |
| α-SMA | 14395-1-AP | Proteintech |
| MMP-2 | 10373-2-AP | Proteintech |
| MMP-9 | 10375-1-AP | Proteintech |
| TIMP1 | ab61224 | Abcam |
| Collagen I | ab34710 | Abcam |
| phospho-Smad3 | ab52903 | Abcam |
| Smad3 | 9523 | Cell Signaling Technology |
| Fibronectin | ab2413 | Abcam |
| TGF-β1 | 21898-1-AP | Proteintech |
| GAPDH | 60004-1-Ig | Proteintech |
| HRP-conjugated goat anti-rabbit IgG | SA00001-2 | Proteintech |
| HRP-conjugated goat anti-mouse IgG | SA00001-1 | Proteintech |

**Table S2** Antibodies used for Western blot

| Antibody | Cat No. | Supplier |
| --- | --- | --- |
| E-cadherin | 14472 | Cell Signaling Technology |
| vimentin | 5741 | Cell Signaling Technology |
| α-SMA | 14395-1-AP | Proteintech |
| collagen I | ab34710 | Abcam |
| phospho-Smad3 | ab52903 | Abcam |
| TGF-β1 | 21898-1-AP | Proteintech |

**Table S3** Primer pairs used for qRT-PCR

| Name | Sequences (5’ to 3’) |
| --- | --- |
| GAPDH *Homo* | F: AGAAGGCTGGGGCTCATTTG |
|  | R: AGGGGCCATCCACAGTCTTC |
| Collagen I *Homo* | F: CCCGGGTTTCAGAGACAACTTC |
|  | R: TCCACATGCTTTATTCCAGCAATC |
| α-SMA *Homo* | F: AGGCACCCCTGAACCCCAA |
|  | R: CAGCACCGCCTGGATAGCC |
| Fibronectin *Homo* | F: CGGTGGCTGTCAGTCAAAG |
|  | R: AAACCTCGGCTTCCTCCATAA |
| GAPDH *Mus* | F: CATCACTGCCACCCAGAAGACTG |
|  | R: ATGCCAGTGAGCTTCCCGTTCAG |
| TGF-β1 *Mus* | F: CCTGAGTGGCTGTCTTTTGA |
|  | R: CGTGGAGTTTGTTATCTTTGCTG |

**Table S4** Antibodies used for Immunofluorescence

| Antibody | **Cat No.** | Supplier |
| --- | --- | --- |
| phospho-Akt | 4060 | Cell Signaling Technology |
| E-cadherin | 14472 | Cell Signaling Technology |
| vimentin | 5741 | Cell Signaling Technology |
| α-SMA | 14395-1-AP | Proteintech |
| Collagen I | ab34710 | Abcam |
| phospho-Smad3 | ab52903 | Abcam |
| Goat Anti-Rabbit IgG | A23220 | Abbkine |
| Goat Anti-Mouse IgG | A23410 | Abbkine |


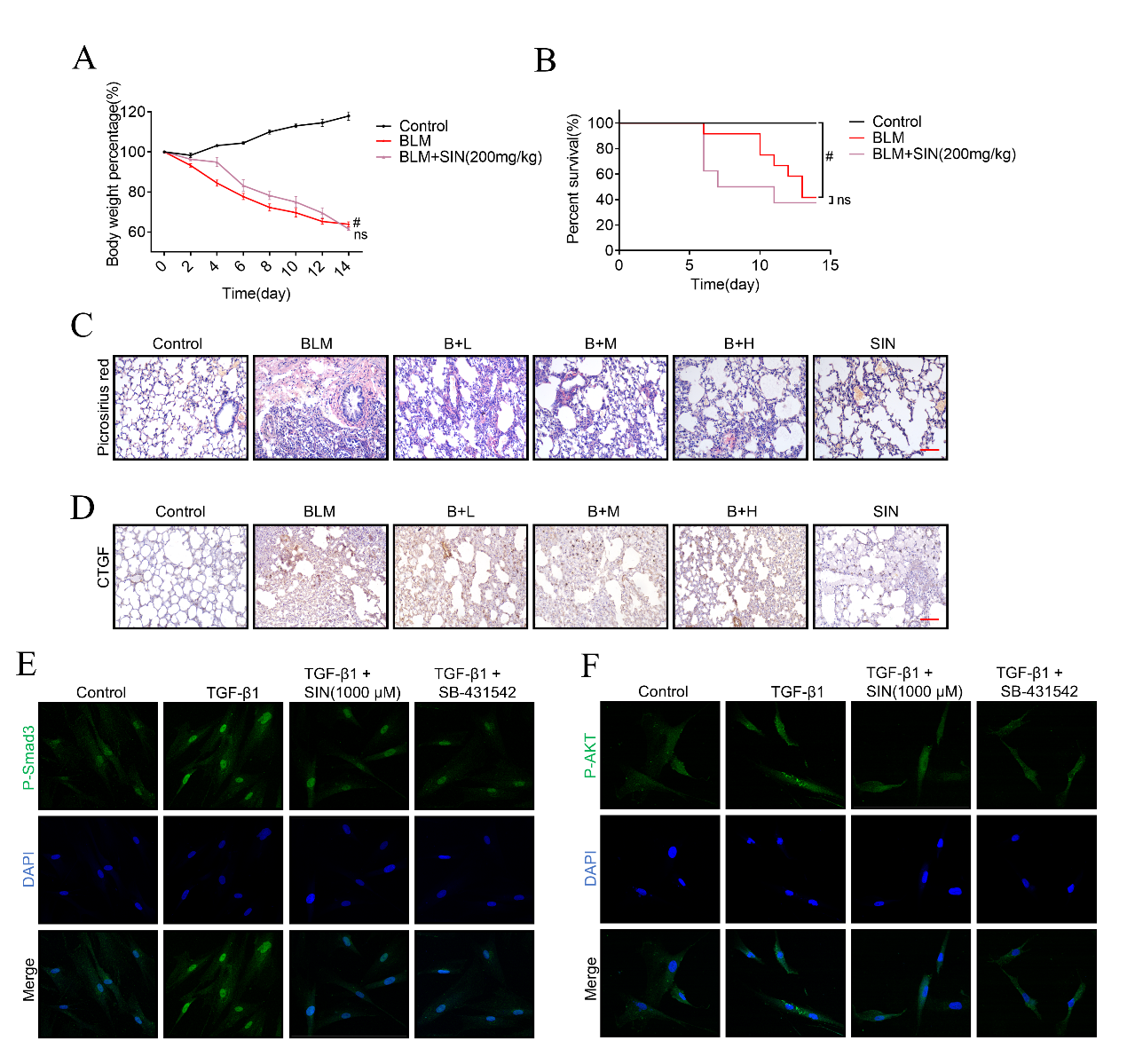


**Fig. S1** **(A, B)** The BLM-induced mouse model was treated by intraperitoneal injection of 200mg/kg SIN per day. Figure A shows the weight change curve and Figure B shows the survival rate curve. n = 6 mice per group. **(C)** Representative micrographs of Sirius red staining in paraffin-embedded lung tissue sections (200x magnification). **(D)** Immunohistochemistry showed the effect of SIN on the expression of CTGF in lung tissue of BLM-induced pulmonary fibrosis mice on the 14th day (200x magnification). **(E)** The representative images of cells in each group were immunostained after treatment, which showed P-smad3 (green) and DAPI (blue) stained nuclei in cells. **(F)** The representative images of cells in each group were immunostained after treatment, which showed P-AKT (green) and DAPI (blue) stained nuclei in cells. n = 3. Scale bar: 5 μm. Results are shown as mean ± SEM. Statistical analysis was performed by one-way analysis of variance. # *p*<0.05, compared with control. ns, not significant, compared with TGF-β1.
